# Breast conserving surgery with preservation of the nipple-areola complex as a feasible and safe approach in male breast cancer: a case report

**DOI:** 10.1186/1752-1947-2-126

**Published:** 2008-04-28

**Authors:** Sophocles Lanitis, George Filippakis, Ragheed Al Mufti, Dimitri J Hadjiminas

**Affiliations:** 1 Breast Care Unit, Mary Stanford Wing 5th Floor, St Mary's NHS Trust, Praed Street, London W2 1NY, UK

## Abstract

**Introduction:**

Breast cancer in men is rare. The evidence about treatment has been derived from data on the management of the disease in women. The usual treatment is for male patients to undergo modified radical mastectomy. There is insufficient experience of breast conserving surgery with preservation of the nipple. The management of patients who demand such an approach for personal reasons remains a challenge for both the surgeon and oncologist.

**Case presentation:**

A 50-year-old man with a breast cancer was successfully managed with breast conserving surgery with nipple preservation combined with axillary clearance and postoperative radiotherapy, chemotherapy and hormone treatment. Since there are no similar cases in the literature, we discuss the feasibility, safety and possible indications of such an approach.

**Conclusion:**

Despite the limited indications and evidence about the safety and efficacy of breast conserving surgery with nipple preservation in men with breast cancer, it is a feasible approach if other options are declined by the patient. More studies are necessary to reach firm conclusions about the safety of such an approach.

## Introduction

Male breast cancer (MBC) is a rare disease and accounts for less than 1% of breast cancers but incidence seems to be increasing [1-6]. Owing to the small number of cases, management of MBC is based on evidence derived from data analysis of female breast cancer (FBC) patients and on retrospective studies of a limited number of MBC patients [2,3,5]. There is little experience of breast conserving surgery (BCS) with nipple preservation, as usually there is no interest for the treatment from either the surgeon or patient. Therefore, management of those patients who demand such an approach remains a challenge for the treating physicians.

## Case presentation

A 50-year-old man was referred to the breast unit presenting with a month's history of a suspicious lump in his left breast. He had no family history of cancer. From his medical history, the only remarkable finding was hepatitis B 30 years previously and genital herpes for which he was taking Acyclovir. He had a history of smoking (30 packs per year).

Clinically, he had a lump centrally in the left breast, at a 6 o'clock position, with skin tethering and mild inversion of the nipple. Ultrasound (US) and a mammogram demonstrated a 1 cm suspicious lesion, which was found to be cancer on both fine needle aspiration (FNA) and core biopsy. The tumour was a grade 2 invasive ductal carcinoma (IDC). Investigations did not show any distant metastases. The patient was offered modified radical mastectomy and sentinel node biopsy (SNB). The patient declined any operation that would not preserve the nipple and insisted on having BCS. After discussion with the oncologists and patient about the risk of recurrence, we proceeded with BCS with nipple preservation and SNB. The sentinel node was involved and level III axillary clearance was performed. Overall, one out of nine dissected lymph nodes was positive. Our well-established protocols for wide local excisions rely on 5 mm pathological clear margins rather than negative margins [7]. Therefore, we do not use frozen sections to assess the surgical margins. The specimen weighed 19 g and included a 0.7 × 0.7 × 1 cm grade 2 IDC with an intermediate grade ductal carcinoma *in situ *(DCIS), comprising 5% of the tumour mass. The tumour was positive for oestrogen (ER) and progesterone receptors (PgR). The DCIS was present 0.2 cm from the superficial margin and all other margins were more than 0.8 cm.

The patient had four cycles of chemotherapy (Doxorubicin 100 mg and Cyclophosphamide 1000 mg) and adjuvant chest wall radiotherapy (50 gray in 25 fractions), which he tolerated well. He was commenced on Tamoxifen 20 mg once a day for 5 years and then switched to Letrozole 2.5 mg. Repeated followup clinical examinations, mammograms, breast US, bone scans and liver US showed no evidence of disease. Eight years after the operation, the mammogram showed microcalcifications in the ipsilateral breast and he underwent diagnostic biopsy of the area, which showed fibrofatty tissue with focal stromal calcifications without features of malignancy.

## Discussion

MBC behaves in a way similar to FBC in postmenopausal women [6]. Unlike FBC, there is only one peak at 67–71 years of age [2,4,6]. Family history, genetic factors (for example, *BRCA *gene carriers, *AR *and *CYP17 *gene mutation, Klinefelter syndrome, Cowden syndrome), exogenous oestrogen administration and testicular anomalies are among the risk factors [1-4,6], while radiation, obesity and alcohol use are proposed but not widely accepted as risk factors [2,3,6]. There is no proven association between gynaecomastia and MBC [4,6]. Histologically, more than 85% of tumours are of the invasive ductal type [4,6]. Furthermore, over 90% of MBC express ER while 81% express PgR [5,8]. C-erb-B2 is less likely to be expressed (about 5%) [2,3]. In men, 20% of the circulating estrogen is produced by the testis while about 80% results from peripheral aromatisation of androgens [3,9]. The usual presentation is a palpable painless lump with or without skin changes or nipple involvement but often diagnosis is delayed [4,6]. The sensitivity of the mammogram is reported to be 92% while specificity is 90% [6]. Breast US can be used to evaluate the tumour in the same way as in women [2,4,10]. The prognosis depends on tumour size, grade and extent of lymph node involvement in the same way as in FBC [2,6]. Overall survival rate when corrected for age is similar to that of FBC [2,6].

Traditionally patients with MBC undergo modified radical mastectomy with either SNB or axillary node clearance (ANC) [2,3,6]. Despite the lack of firm evidence about the safety of SNB, increasingly there is an acceptance of the technique and its use [2,6]. Radiation therapy seems to prevent local recurrence but it is not known whether it adds anything to survival. The indications and dose remain the same as in women [2,6]. Ablative techniques aiming to control hormones, including orchidectomy, adrenalectomy and hypophysectomy, have been used in the past but had severe side effects, therefore medical hormone manipulation has been tried [3,5]. For those patients with hormone receptor-positive tumours, there is a clear benefit from the use of Tamoxifen in both disease-free and overall survival [3,5,6,11,12]. There is also a proven effectiveness in those patients with metastatic disease and, therefore, Tamoxifen has been incorporated in the treatment of MBC [2,3,5,11]. There is not sufficient evidence for the use of aromatase inhibitors despite the advances and proven efficacy in FBC and more studies need to be done [2,3,5]. There are case reports supporting a good response to Letrozole [3,13] even after failure of Tamoxifen [3]. There is also some evidence about the effectiveness of adjuvant chemotherapy. One prospective study with a small number of patients (*N *= 24) showed a survival benefit and other studies support this finding [14]. Moreover, retrospective studies show reduction of the risk of local recurrence [2,6,15].

## Conclusion

Despite limited indications and lack of evidence about the safety and efficacy of BCS with nipple preservation in men with breast cancer, it is a feasible approach if other options are declined by the patient. Apparently obtaining good excision margins is the most important predictor of local recurrences as it is for women.

With a case report of only one patient, it is impossible to make any statement about the safety of such an approach and more studies are necessary to reach firm conclusions.

## Abbreviations

ANC: axillary node clearance; BCS: breast conserving surgery; DCIS: ductal carcinoma in situ; FBC: female breast cancer; FNA: fine needle aspiration; IDC: invasive ductal carcinoma; MBC: male breast cancer; SNB: sentinel node biopsy; US: ultrasound.

## Competing interests

The authors declare that they have no competing interests.

## Authors' contributions

SL and GF collected the data and reviewed the literature and case notes and was involved in followup appointments. Furthermore, SL was involved in the active followup and workup of the patient. SL wrote the paper with the assistance of GF. RAM reviewed and edited the initial manuscript. DJH performed the initial operation, and organised the primary management plan of the patient. He supervised the writing and editing of the paper. All the authors have read and approved the final version of the manuscript.

## Consent

Written informed consent was obtained from the patient for publication of this case report and any accompanying images. A copy of the written consent is available for review by the Editor-in-Chief of this journal.
